# Tooth Discoloration and Solubility of Zinc Oxide Eugenol Combined with Different Concentrations of Nano-Curcumin: An *in vitro* Study

**DOI:** 10.30476/dentjods.2022.92933.1687

**Published:** 2023-06-01

**Authors:** Rasoul Sahebalam, Hossein Bagheri, Hamid Jafarzadeh, Hamide Khodkari, Shabnam Ganjehzadeh

**Affiliations:** 1 Dental Research Center, Mashhad University of Medical Sciences, Mashhad, Iran; 2 Dental Materials Research Center, Mashhad University of Medical Sciences, Mashhad, Iran; 3 Private Practice, Mashhad, Iran; 4 Dept. of Periodontics, Dental Research Center, Mashhad University of Medical Sciences, Mashhad, Iran

**Keywords:** Nanomicelle curcumin, Zinc Oxide Eugenol, Metapex, Solubility, Discoloration

## Abstract

**Statement of the Problem:**

Due to the unfavorable properties of eugenol, the eugenol content can be reduced to improve the properties of zinc oxide eugenol (ZOE) by making a new combination of nanocurcumin called curcumin pulpal paste (CPP).

**Purpose:**

The aim of this *in vitro* study was to evaluate the solubility and tooth discoloration of three concentrations of CPP compared to ZOE, and Metapex.

**Materials and Method:**

In this *in vitro* study for evaluating of the solubility, five groups including ZOE, Metapex, and three concentrations of CPP (5%, 10%, and 20%) were tested. For evaluating the solubility, the changes in of the weight of samples were measured at 1, 3, 7 and 30 days after initial setting. In order to evaluate the tooth discoloration, 75 bovine maxillary anterior teeth were filled with one of five pulpal pastes. The changes in tooth color were evaluated at 1 hour, 1 week, 1 and 3 months after material placement.

**Results:**

The solubility increased by increasing the nano-curcumin percentage in CPPs. After 30 days, the solubility of the 5%CPP, and ZOE was not significantly different (*p*= 1.000). According to the colorimetric test, after 3 months, the highest discoloration was recorded for 20% CPP (8.45), and the smallest discoloration was registered for Metapex (4.06). The discoloration of 5% CPP, and 10% CPP was similar to ZOE’s color change (*p*> 0.05).

**Conclusion:**

The results of the present study showed that the solubility of pulpal paste has increased with increasing curcumin concentrations. Therefore, pulpal paste with different nanocurcumin concentrations can be used considering the patient's age and the desired time of deciduous tooth loss, and dissolution of pulpal paste. Concerning the discoloration after 3 months, Metapex was the best material and the most discoloration rate was related to 20% CPP, and there was no difference between the 5% CPP, and 10% CPP with ZOE.

## Introduction

There are several root canal filling materials for deciduous teeth, among which zinc oxide eugenol (ZOE) is more widely used and available [ 1 ]. A review study on eugenol in ZOE reported that it has anti-inflammatory and antibacterial properties, as well as cytotoxic effects [ 2 ]. Depending on its concentration and exposure time, possible adverse effects of eugenol include suppression of cellular respiration, toxicity to macrophages and fibroblasts, decreased vasoconstrictive response, inhibition of prostaglandins, and suppressive and enhancing effects on the immune response [ 3 ]. It is highly toxic in cell culture experiments. When occurs in periapical tissue, this toxicity may delay or stop the healing process [ 4 ]. ZOE contains free eugenol, which is released over time and leads to moderate-to-severe inflammatory response. A decrease in eugenol content of ZOE greatly reduces its toxicity [ 5 ].

 One of the disadvantages of a canal filling material is its high solubility. Therefore, it creates a gap between the filling material and the canal wall, which in turn causes microleakage and affects the success of treatment [ 6 ]. However, due to differences in morphology and pattern of deciduous tooth resorption [ 7 ], the filling material used in the treatment of deciduous roots should be soluble to be absorbed or dissolved by resorption of deciduous teeth [ 8 ]. Therefore, the low absorption rate and the tendency to be retained even after deciduous tooth exfoliation is one of the disadvantages of ZOE. There are also reports on anterior cross-bite, palatal eruption, and ectopic eruption of the succedaneous tooth following ZOE pulpectomy of deciduous tooth [ 9 - 14 ].

On the other hand, the results of endodontic treatments should not only focus on the physiological and functional aspects; aesthetic issues should also be considered in deciduous teeth [ 15 - 16 ]. The most important internal discoloration associated with endodontic treatments is breakdown of necrotic pulp, hemorrhage inside chamber pulp, intracanal drugs, and root filling materials [ 17 - 18 ]. Coronal discoloration associated with canal filling materials [ 17 , 19 ] depends on the duration of their contact with the tooth structure and their chromogenic potential [ 20 ].

Curcumin or diferrolylmethane (C21H20O6) is a hydrophobic polyphenol derived from the rhizome of the turmeric (*Curcuma Longa*) [ 21 ]. Overall, the most important biological effects of curcumin are its anti-cancer and antioxidant, antimicrobial (against various microorganisms), and soothing and anti-inflammatory effects [ 21 - 22 ].

3-(4,5-dimethylthiazol-2-yl)-2,5-diphenyl-2H-tetraz-olium bromide (MTT) test showed that curcumin at low concentrations (0.5 and 1 μM) had no toxic effect on the dental pulp stem cells for 24, 48, and 72 hours, but at the concentrations of 5, 10, and 15 μM, curcumin was toxic for the pulpal stem cells. This toxic effect may be induced by modulating oxidative stress parameters in a time- and dose-dependent manner [ 23 ].

Curcumin can be a promising agent for promotion cell viability, proliferation of primary dental pulp fibroblasts, and vital pulp therapy. Cell viability of primary dental pulp fibroblasts to 25%, 50%, and 100% curcumin concentration was 174%, 310%, and 317%, respectively [ 24 ].

Nanocurcumin higher solubility makes stronger antimicrobial properties. Nanocurcumin exerts its antibacterial effect through penetration into the cell wall and complete rupture of the wall, resulting in bacterial death [ 25 ]. In addition, the anti-inflammatory properties of curcumin increase with increasing its permeability [ 26 ]. Due to its antimicrobial, antioxidant, and anti-inflammatory characteristics, curcumin has been successfully used as a scaffold for the attachment and proliferation of human dental pulp stem cells, and it also could induce mineralization in human dental pulp stem cells, which is essential for healing and repairing the injured dentin-pulp complex [ 27 ]. 

Due to the unfavorable properties of eugenol and the favorable properties of nanocurcumin, we decided to reduce the eugenol content and improve properties of the pulpal paste, by making a new combination of nanocurcumin called curcumin pulpal paste (CPP).

In this study, the solubility and discoloration of different curcumin nano-micelle concentrations (Sina Curcumin) as a new compound were compared with conventional ZOE, which is currently the most common root filling material in the United States [ 28 ]. In Sina curcumin, all curcumin is trapped in the hydrophobic part of the curcumin nano-micelle. These spherical nano-micelles have a particle size of about 10 nanometers that increases curcumin water-solubility by more than 100,000 times and increase its digestive absorption [ 25 ].

Our null hypothesis would be that CPP, ZOE, and Metapex do not differ in solubility and tooth discoloration. 

## Materials and Method

This research has been approved by the Ethics Committee of Mashhad University of Medical Sciences, with the code 1394.222IR.mums.sd.REC. The final sample size was calculated 75 for 5 study groups (n=15 per group) for each time period (4 time periods) in the solubility test considering α =0/05 and β =0/1[ 29 ]. The sample size of the discoloration test was 75 samples [ 30 ].

### Solubility test of pulpal pastes

**Figure 1:**
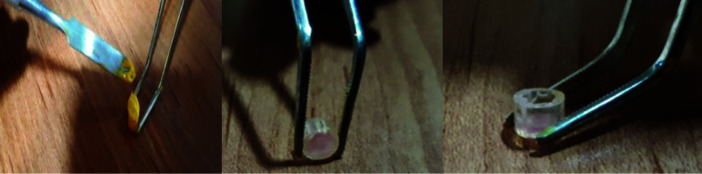
Polyvinyl chloride (PVC) cylinders filling with curcumin pulpal paste (CPP)

In this study, to determine the solubility, the percentage of change in material weight was determined. First, 75 cylinders made of polyvinyl chloride (PVC) with predefined dimensions (thickness= 1mm, diameter= 2.5mm, and height= 3mm) were prepared (Figure 1), and adhesive waxes were used to seal one side of them. The cylinders were divided into 5 groups (n=15 per group). Pulpal pastes were provided by combining equal percentages of zinc oxide (65% ZnO) with hand spatula, and increasing percentages of nanocurcumin (Sina-curcumin, Exir Nano Sina Co., Tehran, Iran) and decreasing percentages of eugenol (Table 1). The material was transferred into a syringe and the dental molds were filled with the filling materials. The molds were first placed along with wet cotton balls at room temperature for one day and then in an incubator (Shin Saeng, model SSI 202, Korea) at 37°C and saturated moisture for the other 5 days [ 29 ]. Samples were taken out of the incubator and excess material was removed with sandpaper. Prior to initial weighing, the samples were placed in a desiccator (Gilson, model MA-203, United States) for 24 hours. Then each sample was weighed with a 0.0001gr precision scale (Denver SI-114, Bohemia, New York) twice, and the average weigh was recorded as the initial weight. Fifteen samples of each pulpal paste were removed from distilled water after 24 hours and re-weighed in a desiccator after overnight dehumidification. All the above steps were repeated for new samples at periods of 72 hours, one week, and four weeks. The percentage of weight change at different time intervals was calculated and considered as the solubility of pulpal pastes.

**Table 1:** Study groups and their components in solubility and colorimetric tests

Group	Type of pulpal paste	Amount of powder	Amount of liquid		Commercial brand
Experimental	5%CPP*	65% ZnO**	5% Curcumin	30% Eugenol	Zinc oxide 99.86%- Golchadent Eugenol BP 100%- Kemdent Curcumin nanomicelle (Sina Curcumin)
	10%CPP	65% ZnO	10% Curcumin	25% Eugenol	
	20%CPP	65% ZnO	20% Curcumin	15% EugenolControl	
Control	ZOE***	65% ZnO	35% Eugenol		
Control	Calcium hydroxide with Iodoform	Available in 2.2 g syringes and disposable tips			BIOMED (Metapex)

* Curcumin pulpal paste

** Zinc oxide

** Zinc oxide eugenol

### Pulpal paste discoloration test

**Figure 2:**
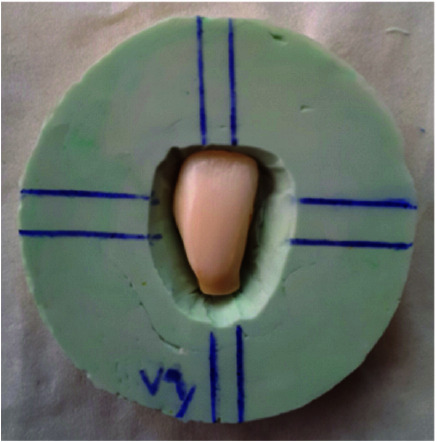
Bovine maxillary anterior tooth molded in a polyethylene ring

**Figure 3:**
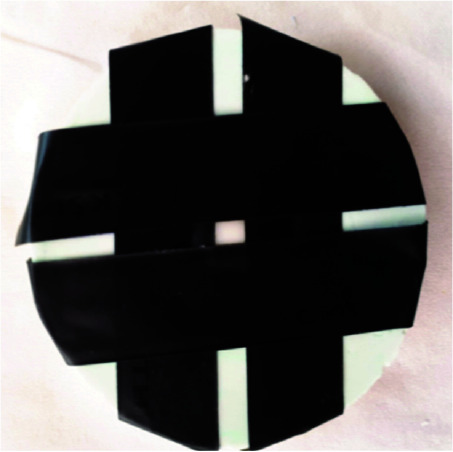
The labial surface of the tooth except one ninth of middle part were covered with black tape

**Figure 4:**
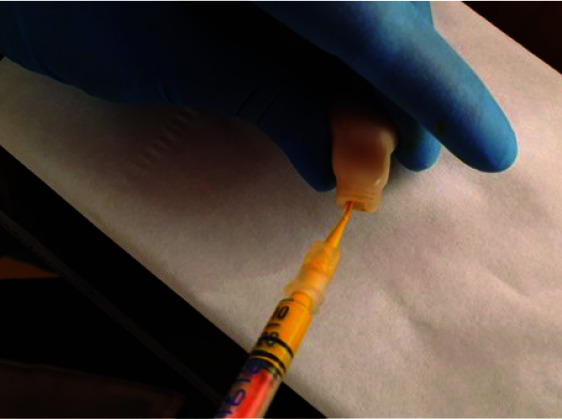
Injecting pulpal paste into the pulp chamber with an insulin syringe

Extracted bovine teeth were used to measure discoloration. These extracted bovine maxillary anterior teeth were evaluated clinically and radiographically. A total of 75 teeth without caries, internal and external discoloration, fracture, crack, abrasion, and pulpal calcification were selected. Plaques and surface debris were removed using a Cavitron ultrasonic scaler (SKL HE02, model K3, Japan). To reduce the scattering of colorimetry data, the labial surface of each tooth was polished with sandpapers (1000, 1500, 2000, 2500, 3000 grit) (STARCKE, Germany), respectively. The teeth roots were cut one millimeter below the cemento-enamel junction (CEJ) using discs and handpieces. The pulp chamber and its horns underwent mechanical and chemical debridement by K-file No. 45 to 80, 2.5% hypochlorite solution, and normal saline. The teeth were divided into 5 groups (n=15 samples) to compare the discoloration of pulpal pastes (Table 1). Each tooth was placed in the center of a polyethylene ring 1cm in height and 10 cm in diameter and molded with a silicone-based high viscosity impression material (Speedex, Coltene Whaledent GmbH, Switzerland) (Figure 2). For the initial colorimetry, each tooth was placed inside its mold and all parts of the labial surface of the tooth except one ninth of middle part were covered with black tape (Figure 3). Colorimetry was performed with a colorimeter (CR-400 Chroma Meter, Konica Minolta Sensing, Japan) in the middle ninth of the buccal surface and was recorded as tooth color at time zero. Each of the pastes was mixed according to the values mentioned in Table 1 and transferred to an insulin syringe with a disposable plastic tip. The pulp chamber of each tooth was dried with dry air for three seconds and pulpal paste injected into it at the CEJ level (Figure 4), and its end was well sealed with light cured glass ionomer (GC-Fuji II LC Gold, Japan). The samples were place in 6-well plates to create saturated moisture and filled with distilled water. The container lid was sealed with laboratory film and transferred to an incubator at 37°C. Subsequent colorimetry assessments were performed at one hour, one week, one month, and three months after setting by repositioning the teeth in the pre-prepared molds in one middle ninth. To prevent the bacterial growth, distilled water in plates containing dental samples were replaced every 3 days.

During the colorimetry, the parameters L, a, and b were examined, and finally ∆E was calculated:

$\mathrm{∆E}={\left[(\mathrm{∆L})+({\mathrm{∆a}}^{2})+({\mathrm{∆b}}^{2})\right]}^{1/2}$

Shapiro-Wilk test was used to evaluate the normality of data distribution. ANOVA test was used in the case of variables with normal data distribution, and Kruskal Wallis and Friedman test were used for other data. The significant level was set on p˂ 0.05. 

## Results

In the present study, 75 polyvinyl chloride (PVC) molds containing different pulpal pastes for each time interval were used to investigate the solubility. To investigate the discoloration of the pulpal pastes, 75 anterior crowns severed from the bovine maxilla were used.

### Solubility test results

The mean and standard deviation (SD) of weight percentage decrease of the samples by pulpal paste type and time is given in Table 2. According to Table 2, the lowest solubility was related to Metapex on all days and the highest solubility was for 20%CPP. Solubility increased significantly in all groups from day 1 to day 30 (*p*< 0.001). The solubility increased by increasing the nano-curcumin percentage in CPPs. After 30 days, the solubility of the 5%CPP, and ZOE was not significantly different (1.000). 

**Table 2:** Mean and standard deviation of reduced weight percentage of samples by pulpal paste type and time

Group		ZOE**	Metapex	5% CPP*	10% CPP	20% CPP	Test result
Number		15	15	15	15	15	
Day 1	Mean	1.46	0.38	1.44	4.44	8.78	Χ^2^ = 64.93
	SD***	0.37	0.55	0.24	0.53	0.45	*p*< 0.001
Day 3	Mean	2.99	0.50	3.97	7.06	12.09	F = 831.38
	SD	0.32	0.20	0.40	0.54	1.08	*p*< 0.001
Day 7	Mean	4.03	1.31	6.66	11.11	14.98	Χ^2^ = 70.87
	SD	0.49	0.39	0.89	0.65	1.37	*p*< 0.001
Day 30	Mean	6.66	3.01	7.35	11.49	15.83	Χ^2^ = 68.56
	SD	0.79	0.56	0.54	0.66	0.90	*p*< 0.001
Test result		F* = 255.09	Χ^2^ = 46	F = 330.89	F= 472.92	Χ^2^ = 51	
		*p*< 0.001	*p*< 0.001	*p*< 0.001	*p*< 0.001	*p*< 0.001	

The result of One-way ANOVA test

* Curcumin pulpal paste

** Zinc oxide eugenol

*** Standard deviation

### Results of colorimetric tests

Mean and standard deviation of discoloration by pulpal paste type and time are given in Table 3 (0= before setting, 1= one hour after setting, 2= one week later, 3= one month later, 4= three months later). Metapex was the best material in terms of discoloration rate after 3 months (4.06%) and the most discoloration was related to 20% CPP (8.45%) and there was no difference between 5% CPP, and 10% CPP with ZOE (*p*> 0.05) (Table 3). Among the curcumin-containing pastes, the least discoloration rate after 1 hour was related to the 5% CPP, which was not significantly different from Metapex (*p*= 1.000), and was significantly less than ZOE (*p*= 0.019) (Table 4). Significant discoloration occurred in the first week after setting (*p*= 0.002). There was no significant difference between groups in terms of discoloration after one week. Increasing the curcumin concentration from 5 to 20% increases the discoloration rate, but it is not statistically significant.

**Table 3:** Mean and standard deviation of discoloration of samples by pulpal paste type and time

Group	Number		∆E 01	∆E02	∆E03	∆E 04	∆E12	∆E13	∆E14	∆E23	∆E24	∆E34	Friedman Test result
ZOE**	15	Mean	9.90	6.52	6.29	4.80	6.89	7.85	7.17	3.75	3.77	3.66	Χ^2^ =33.05
		SD	4.12	3.45	2.76	1.52	4.48	5.04	4.99	2.17	2.94	2.32	*p*< 0.001
Metapex	15	Mean	4.02	4.90	5.20	4.06	3.59	3.65	3.48	3.85	3.38	2.51	Χ^2^=15.26
		SD	2.15	2.62	2.47	1.54	2.11	2.33	2.14	3.35	2.17	2.32	*p*= 0.084
5% CPP*	15	Mean	5.66	6.45	6.59	6.61	3.98	4.93	5.50	3.35	3.18	3.69	Χ^2^=39.14
		SD	2.29	2.65	2.45	2.13	3.23	2.58	2.65	1.89	1.96	2.24	*p*< 0.001
10% CPP	15	Mean	7.18	8.34	8.12	7.36	3.64	3.68	3.82	3.93	4.41	3.00	Χ^2^=48.51
		SD	3.82	4.18	3.72	3.62	2.76	3.59	2.32	2.50	1.82	1.91	*p*< 0.001
20% CPP	15	Mean	7.10	9.35	8.69	8.45	6.10	6.56	5.55	3.46	4.12	3.58	Χ^2^=67.20
		SD	3.0	2.7	2.6	2.5	3.06	3.16	2.79	1.95	2.33	2.31	*p*< 0.001
Result of Kruskal		Χ^2^	22.34	16.72	14.86	28.86	12.18	15.18	10.89	0.36	4.30	4.59	
		P	<0.001	0.002	0.005	<0.001	0.016	0.004	0.028	0.986	0.367	0.332	

(0= before setting, 1= one hour after setting, 2= one week later, 3= one month later, 4= three months later).

* Curcumin pulpal paste

** Zinc oxide eugenol

**Table 4:** Pairwise comparison of discoloration

Group		*p* Value						
		∆E 01	∆E 02	∆E 03	∆E 04	∆E 12	∆E 13	∆E 14
ZOE**	Metapex	< 0.001	1.000	1.000	1.000	0.259	0.65	0.123
	5%CPP	0.019	1.000	1.000	0.334	0.227	1.000	1.000
	10%CPP	0.730	1.000	1.000	0.203	0.158	0.022	0.151
	20%CPP	1.000	0.158	0.232	0.002	1.000	1.000	1.000
Metapex	5%CPP	1.000	1.000	1.000	0.005	1.000	1.000	0.530
	10%CPP	0.059	0.070	0.063	< 0.001	1.000	1.000	1.000
	20%CPP	0.034	0.001	0.004	< 0.001	0.348	0.162	0.530
5%CPP*	10%CPP	1.000	1.000	1.000	1.000	1.000	1.000	0.629
	20%CPP	1.000	0.158	0.510	1.000	0.307	1.000	1.000
10%CPP	20%CPP	1.000	1.000	1.000	1.000	0.217	0.060	0.629

(0= before setting, 1= one hour after setting, 2= one week later, 3= one month later, 4= three months later).

* Curcumin Pulpal Paste

* Zinc Oxide Eugenol

## Discussion

The results of this study showed that the pulpal paste solubility has increased by increasing curcumin concentration. The weight change on day 1 in the 10%CPP, and 20% CPP groups was 4.44% and 8.78%, respectively, which is more than the amount defined by the standard No.57 (2000) & No.30 (2001) ANSI / ADA (less than 3%). ZOE, Metapex, and 5% CPP groups met the above standard. On the other hand, there was no significant difference between 5% CPP with the other two control groups in terms of solubility after 24 hours. On the third day, increasing curcumin concentration had an evident effect in increasing the solubility.

Due to this study results, increasing curcumin concentration in the structure of curcumin-containing pastes can cause disintegration of different components of the material and increase its solubility. Therefore, according to the patient's age and the desired time of tooth loss and pulpal paste dissolution, pulpal paste with different nanocurcumin concentrations can be used.

In the case of discoloration, the acceptable threshold for ∆E is 3.7 units, which, if exceeded, can be clinically perceived by the human eye. Below that, color matching occurs and the changes become imperceivable [ 31 ]. Among the curcumin-containing pastes, the least discoloration rate after 1 hour was related to the 5% CPP, which was not significantly different from Metapex, and was significantly less than ZOE. Significant discoloration occurred in the first week after setting. There was no significant difference between groups in terms of discoloration after one week, which could be due to the infiltration of materials from the dentinal tubules and exchange with the aqueous medium and the need to reach balance. Considering the effect of time on the discoloration of each material, it can be concluded that curcumin-containing pastes do not change significantly with increasing time, which can be due to different setting properties or balance and exchange with the environment. Increasing the curcumin concentration from 5 to 20% increases the discoloration rate, but it is not statistically significant, which can be due to the maximum color changes caused by this material. Meta-pex was the best material in terms of discoloration rate after 3 months (4.06%), the most discoloration was related to 20% CPP (8.45%), and there was no difference between 5% CPP and 10% CPP with ZOE (*p*> 0.05).

Wilson [ 32 ] stated that the most appropriate method to investigate the vanishing of the root canal sealer is to weigh them before and after placing them in an aqueous mIn the present study, in order to investigate the solubility of pulpal pastes, we have investigated their weight change over time. In the present study, a scale with precision of 0.0001kg was used, which is similar to the one used in studies by Maura *et al*. [ 33 ], and Danesh *et al*. [ 34 ].

Hamed *et al*. [ 35 ] evaluated the solubility of Endofil sealer at 1, 7, 14, 28, and 56-day time intervals. In most studies, the solubility was assessed 24 hours after the material was set, which is the standard approved by the ADA [ 36 - 37 ]. Considering that solubility changes over time, it is verified that the ZOE solubility would increase over time [ 35 ]. Therefore, in the present study, in addition to 24 hour-time interval, solubility assessment was carried out at 3, 7 and 28-day time intervals in order to further investigate the solubility of pulpal paste and to identify changes in their solubility over time. The present study showed that the solubility of all groups increased over time, although 5% CPP is approved by A-DA and had even less solubility than ZOE in distilled water after 24 hours, its solubility was higher than ZOE from day 3 onwards, but this increase was still not significant. There was also no significant increase in solubility of all three curcumin concentrations from day 7 onwards.

Comparing the solubility of three materials (Apexcal, Zical, and ZOE) by method of changing the sample weight, Amro M. *et al*. [ 38 ] found that the mean solubility of the two experimental groups was lower than the ZOE control group, but still the mean solubility of all study groups was less than 3% after 24 hours according to ADA/ ANSI NO57 & 30. In our study, the solubility of 5% CPP was only less than ZOE control groups and more than the Metapex group. According to the ADA standard, the solubility of all three groups was below 3% after 24 hours, but the solubility in the 10% CPP, and 20% CPP groups was more than 3% and the mean solubility in these groups was also significantly higher than the control groups [ 39 ]. 

In this study, the bovine anterior teeth with similar morphology to human teeth were used to determine the discoloration rate. There is no significant difference between bovine and human teeth in terms of dentin tubules [ 40 ]. Although, the selection criteria for deciduous tooth filling materials, concerning differences in root morphology, optimal resorption pattern, and radiopacity, differ from permanent teeth, we can compare results of studies carried out on them [ 7 ]. To obtain generalized results, the use of permanent teeth is recommended in future studies.

In order to reduce the scattering of the colorimetry data, teeth surfaces were polished using five grades of sandpapers (rough to fine) before colorimetric assessment. Also, to keep the device angle constant during colorimetry for each tooth, a separate putty mold was prepared and colorimetric assessment was performed only in the one the middle ninth of the buccal surface.

Colorimetric assessment is carried out with a visual technique or instruments such as a spectrophotometer or calorimeter. Unlike visual assessment, which is most commonly used in dental treatment, the ideal colorimetric assessment method should be reliable, repeatable, and easy to compare measured colors [ 41 ]. In this study, the colorimeter device (CHOROM METER Konica Minolta CR-400) was used, which has all the above characteristics.

Van der Burgt *et al*. [ 17 ] measured tooth discoloration with the naked eye and based on the judgment of two calibrated individuals. Partovi *et al*. [ 19 ], Parsons *et al*. [ 20 ], and Zare Jahromi *et al*. [ 42 ] first took photographs of the teeth, compared with photographs taken at different times, and analyzed with computer software. These methods are less reliable than the method used in the present study. Akbari *et al*. [ 43 ] measured the mineral trioxide aggregate (MTA) induced tooth discoloration using a calorimeter. 

In a study, Zare Jahromi *et al*. [ 42 ] compared the two experimental groups (ZOE and AH26) and the two blood control groups without material setting. They compared images taken from the labial surface of 50 anterior maxillary human teeth at different times and analyzed them using a software. They found significantly higher discoloration in AH26 after 4 months compared to ZOE. In the present study, the discoloration of all three curcumin concentrations was more than ZOE, but discoloration rate was statistically significant on in 20%CPP.

Samantha R *et al*. [ 44 ] compared and examined the discoloration of deciduous root canal filling materials, including ZOE, Vitapex^®^, and Calen^®^/Zo (calcium hydroxide and zinc oxide), an MTA-based material, and empty control group, in 75 bovine enamel-dentin blocks by spectrophotometer and Easy-shade device under standard conditions for 9 months. They stated that the lowest discoloration rate related to the MTA-based material and was not significantly different from the control group. The highest discoloration rate was related to ZOE, which was statistically significant from the control group (*p*= 0.018). In the present study, the lowest discoloration rate after 3 months was related to Metapex^®^, and there was no significant difference between 5% CPP, and 10% CPP groups with ZOE. The highest discoloration rate was related to 20%CPP, which was also statistically significant from the control group.

The application of different nanocurcumin concentrations and the use of two different control groups were the strengths of this study. Due to limited information on solubility properties and discoloration of canal filling materials, further *in vitro*, *ex vivo*, and *in vivo* studies with larger sample sizes and longer follow-up periods are required to further ensure the clinical and radiographic success of the studied material (CPP) and investigate its usefulness as a canal filling material for deciduous teeth. It is also suggested to investigate other properties of the material, including dimensional modification, canal wall binding, flow, aging, and leakage.

## Conclusion

The results of the present study showed that the solubility of pulpal paste has increased with increasing curcumin concentrations. Therefore, pulpal paste with different nanocurcumin concentrations can be used according to the patient's age and the desired time of tooth loss and dissolution of pulpal paste. With regard to discoloration after 3 months, calcium hydroxide with Iodoform (Metapex) was the best material and the most discoloration rate was related to 20%CPP. 

## Acknowledgement

This work was supported by Mashhad University of Medical Sciences, Iran.

## Conflict of Interest

Authors declare that there are no conflicts of interest in this study. 
